# Supplementary material for: Activation of hedgehog signaling in mesenchymal stem cells induces cartilage and bone tumor formation via Wnt/β-Catenin
Source: eLife. 2019 Sep 4;8:e50208. doi: 10.7554/eLife.50208 (PMC6764825; doi:10.7554/eLife.50208)
Supplement: Supplementary file 1. [file elife-50208-supp1.docx]

| **Key Resources Table** | | | | |
| --- | --- | --- | --- | --- |
| **Reagent type (species) or resource** | **Designation** | **Source or reference** | **Identifiers** | **Additional information** |
| Genetic reagent (M. musculus) | Prrx-creERT | PubMed: 19538944 | RRID:  MGI:J:151681 | Dr. Shunichi Murakami  (Case Western Reserve University, Cleveland, USA) |
| Genetic reagent (M. musculus) | Gli1-CreERT | PubMed: 15315762 | RRID: MGI:3053957 | Jackson Laboratory  (Stock No: 007913) |
| Genetic reagent (M. musculus) | Rosa-Ai14 | PubMed: 20023653 | RRID:  MGI: J:155793 | Jackson Laboratory  (Stock No: 007914) |
| Genetic reagent (M. musculus) | Ptch1^f/f^ | PubMed: 12872247 | RRID:  MGI: J:85040 | Jackson Laboratory  (Stock No: 030494) |
| antibody | anti-Collagen I α (Rabbit polyclonal) | Abcam | Cat# ab21286, RRID:AB_446161 | IF(1:100) |
| antibody | anti-Collagen II (Rabbit polyclonal) | Abcam | Cat# ab34712,  RRID:AB_731688 | IF(1:100) |
| antibody | anti-Collagen X (Rabbit polyclonal) | Abcam | Cat# ab58632, RRID:AB_879742 | IF(1:100) |
| antibody | Purified Rat Anti-Mouse CD31 (Rat monoclonal) | BD Biosciences | Cat# 550274, RRID:AB_393571 | IF(1:100) |
| antibody | anti-Vimentin (Rabbit monoclonal) | Abcam | Cat# ab92547, RRID:AB_10562134 | IF(1:100) |
| antibody | anti-Perilipin-1 (Rabbit polyclonal) | Abcam | Cat# ab3526, RRID:AB_2167274 | IF(1:100) |
| antibody | anti-Ki67 (Rabbit polyclonal) | Abcam | Cat# ab15580, RRID:AB_443209 | IF(1:100)  IHC(1:200) |
| antibody | Phospho-Akt (Ser473) (Rabbit monoclonal) | Cell Signaling Technology | Cat# 4060, RRID:AB_2315049 | IHC(1:100) |
| antibody | Phospho-Smad1/5/8 (Rabbit monoclonal) | Cell Signaling Technology | Cat# 13820, RRID:AB_2493181 | IHC(1:100) |
| antibody | Anti-FSP1 (Rabbit polyclonal) | Abcam | Cat# ab27957, RRID:AB_2183775 | IHC(1:200) |
| antibody | GLI1 (Rabbit polyclonal) | SAB | Cat# 43926 | IHC(1:100) |
| antibody | anti-beta Catenin (Rabbit monoclonal) | Abcam | Cat# ab32572, RRID:  AB_725966 | IHC(1:100)  WB(1;1000) |
| antibody | β-Actin (Mouse monoclonal) | Santa Cruz Biotechnology | Cat# sc-47778 RRID:  AB_2714189 | WB(1:1000) |
| antibody | Phospho-Akt (Ser473) (Rabbit polyclonal) | Cell Signaling Technology | Cat# 9271, RRID:  AB_329825 | IHC(1:100)  WB(1:1000) |
| antibody | Akt (Rabbit polyclonal) | Cell Signaling Technology | Cat# 9272, RRID:  AB_329827 | WB(1:1000) |
| antibody | Phospho-p44/42 MAPK (Erk1/2) (Thr202/Tyr204) (Rabbit monoclonal) | Cell Signaling Technology | Cat# 4377, RRID:  AB_331775 | WB(1:1000) |
| antibody | p44/42 MAPK (Erk1/2) (Rabbit polyclonal) | Cell Signaling Technology | Cat# 9102, RRID:  AB_330744 | WB(1:1000) |
| antibody | Phospho-Smad1 (Ser463/465)/ Smad5 (Ser463/465)/ Smad8 (Ser426/428) (Rabbit polyclonal) | Cell Signaling Technology | Cat# 9511, RRID:  AB_331671 | IHC(1:200)  WB(1:1000) |
| antibody | anti-Smad1 (Rabbit polyclonal) | Cell Signaling Technology | Cat# 9743, RRID:  AB_2107780 | WB(1:1000) |
| antibody | PTCH1 (Rabbit polyclonal) | Proteintech | Cat# 17520-1-AP,  RRID:  AB_2176561 | WB(1:1000) |
| antibody | GLI1 (Rabbit polyclonal) | Cell Signaling Technology | Cat# 2553, RRID:  AB_2294701 | IHC(1:100）WB(1:500) |
| antibody | Phospho-Smad2 (Ser465/467) (Rabbit polyclonal) | Cell Signaling Technology | Cat# 3101, RRID:  AB_331673 | WB(1:500) |
| antibody | Phospho-Smad3 (Ser423/425) (Rabbit monoclonal) | Cell Signaling Technology | Cat# 9520, RRID:  AB_2193207 | WB(1:500) |
| antibody | Smad2/3 (Rabbit polyclonal) | Cell Signaling Technology | Cat# 3102, RRID:  AB_10698742 | WB(1:500) |
| recombinant DNA reagent | pHBLV-CMV-Puro-hPtc1 | This study |  | See ’Materials  and methods |
| sequence-based reagent | RT-qPCR primers | This paper |  | See supplemental Table S1 |
| sequence-based reagent | CHIP primers | This paper |  | See supplemental Tabel S2 |
| peptide, recombinant protein | Recombinant human sonic hedgehog /Shh Protein | R&D systems | Cat# 8908-SH-005 |  |
| commercial assay or kit | PrimeScript RT  reagent Kit | TAKARA | RR037A |  |
| commercial assay or kit | Fast Start Universal SYBR Green Master kit | Roche | 04887352001 |  |
| commercial assay or kit | SimpleChIP® Enzymatic Chromatin IP Kit | CST | #9002 |  |
| commercial assay or kit | nuclear and cytoplasmic protein extraction kit | Beyotime | P0027 |  |
| commercial assay or kit | Cell Counting Kit-8 | Sangon Biotech | E606335 |  |
| commercial assay or kit | Alkaline Phosphatase Kit | Sigma | 85L2-1KT |  |
| chemical compound, drug | IWP-2 | Selleck Chemicals | Cat# S7085 |  |
| chemical compound, drug | FH535 | Selleck Chemicals | Cat# S7484 |  |
| chemical compound, drug | Cyclopamine | Selleck Chemicals | Cat # S1146 |  |
| chemical compound, drug | GANT61 | Selleck Chemicals | Cat# S8075 |  |
| software, algorithm | ImageJ | ImageJ (http://imagej.nih.gov/ij/) |  |  |
| software, algorithm | GraphPad Prism 8 | https://www. graphpad.com | RRID:SCR_015807 | Version 8 |
| software, algorithm | Image Pro Plus | http://www.mediacy.com/ |  | Version 6 |
