# Supplementary material for: Activation of hedgehog signaling in mesenchymal stem cells induces cartilage and bone tumor formation via Wnt/β-Catenin
Source: eLife. 2019 Sep 4;8:e50208. doi: 10.7554/eLife.50208 (PMC6764825; doi:10.7554/eLife.50208)
Supplement: Supplementary file 2. [file elife-50208-supp2.docx]

**Supplemental Table S1: Quantitative PCR Primer sequences used in the study**

| **Gene** | **Forward sequence** | **Reverse sequence** | **Accession Number** |
| --- | --- | --- | --- |
| OSX | ACTCATCCCTATGGCTCGTG | GGTAGGGAGCTGGGTTAAGG | NM_001348205.1 |
| SOX9 | AGTACCCGCATCTGCACAAC | ACGAAGGGTCTCTTCTCGCT | NM_011448.4 |
| MMP13 | TGTTTGCAGAGCACTACTTGAA | CAGTCACCTCTAAGCCAAAGAAA | NM_008607.2 |
| OCN | CAGACAAGTCCCACACAG | GCAGAGTGAGCAGAAAGA | L24431.1 |
| ALP | ATGCCCTGAAACTCCAAA | AGACGCCCATACCATCTC | NM_007431.3 |
| Col2a1 | CCTCAAGGCAAAGTTGGTCCT | CACACGTCTCGGTCATGGTA | NM_031163.3 |
| CEBPα | GGACAAGAACAGCAACGAG | TCACTGGTCAACTCCAGCAC | NM_007678 |
| Acan | GTGGAGCCGTGTTTCCAAG | AGATGCTGTTGACTCGAACCT | [NM_007424.2](https://www.ncbi.nlm.nih.gov/nucleotide/NM_007424.2?report=genbank&log$=nucltop&blast_rank=2&RID=54YC2810015) |
| FABP4 | GATGAAATCACCGCAGACGACA | ATTGTGGTCGACTTTCCATCCC | NM_024406 |
| PPARγ | GGAAGACCACTCGCATTCCTT | GTAATCAGCAACCATTGGGTCA | NM_001127330.2 |
| Adamts5 | GGAGCGAGGCCATTTACAAC | CGTAGACAAGGTAGCCCACTTT | [NM_011782.2](https://www.ncbi.nlm.nih.gov/nucleotide/NM_011782.2?report=genbank&log$=nucltop&blast_rank=1&RID=54VYM4SR014) |
| Hhip1 | GAAGATGCTCTCGTTTAAGCTGC | CCACCACACAGGATCTCTCC | [NM_020259](http://www.ncbi.nlm.nih.gov/entrez/query.fcgi?cmd=Search&db=Nucleotide&term=NM_020259) |
| WNT1 | TCGGCAAGATCGTCAACC | ATGGAGCCTTCGGAGCAG | [NM_021279.4](https://www.ncbi.nlm.nih.gov/nucleotide/NM_021279.4?report=genbank&log$=nucltop&blast_rank=1&RID=55497JA8014) |
| WNR5a | CACGCTATACCAACTCCTCTGC | AATATTCCAATGGGCTTCTTCATGGC | [NM_009524.3](https://www.ncbi.nlm.nih.gov/nucleotide/NM_009524.3?report=genbank&log$=nucltop&blast_rank=1&RID=554CNCSR015) |
| WNT6 | ACGAGCGGATCTCCTCTACG | CGGCACAGACAGTTCTCCT | [XM_006495888.3](https://www.ncbi.nlm.nih.gov/nucleotide/XM_006495888.3?report=genbank&log$=nucltop&blast_rank=1&RID=554H9KRW015) |
| WNT9a | ACACCTGGACGACTCTCCC | CTTGTCACCACACGACTCTGT | [XM_006532855.2](https://www.ncbi.nlm.nih.gov/nucleotide/XM_006532855.2?report=genbank&log$=nucltop&blast_rank=1&RID=554P7M24015) |
| WNT16 | AGAGTGCAACCGGACATCAG | CGTAGCAGCACCAGATAAACTT | [NM_053116.4](https://www.ncbi.nlm.nih.gov/nucleotide/NM_053116.4?report=genbank&log$=nucltop&blast_rank=1&RID=554SPRYK014) |
| Timp3 | GCAAGGGCCTCAATTACCG | AGGCGTAGTGTTTGGACTGATA | [NM_011595](http://www.ncbi.nlm.nih.gov/entrez/query.fcgi?cmd=Search&db=Nucleotide&term=NM_011595) |
| Gli1 | GCACCACATCAACAGTGAGC | GCGTCTTGAGGTTTTCAAGG | [NM_010296.2](https://www.ncbi.nlm.nih.gov/nucleotide/NM_010296.2?report=genbank&log$=nucltop&blast_rank=1&RID=554W9RK9014) |
| Gli2 | CATGGTATCCCTAGCTCCTC | GATGGCATCAAAGTCAATCT | [NM_001081125.1](https://www.ncbi.nlm.nih.gov/nucleotide/NM_001081125.1?report=genbank&log$=nucltop&blast_rank=3&RID=554ZB6GG015) |

**Supplemental Table S2: Primer sequences used for ChIP assays**

| **Gene** | **Predictive binding site** | **Forward sequence** | **Reverse sequence** |
| --- | --- | --- | --- |
| *Wnt5a* | S1: ATACCACACACA | 5'-TAGGACAGCCTCATCACGCA-3' | 5'-ACTTTCGCCCTCCCTCTCTC-3' |
|  | S2: ATACCACACACA |  |  |
| *Wnt6* | S3: TGCCCACACAGG | 5'-CAGGCCCATCCCTCTCTTCA-3' | 5'-GCGCATGTTTCCTAACCCGA-3' |
|  | S4: CATGGGTGGTCA | 5'-GTGCAGCTGTGGAGGGAATG-3' | 5'-AGAGAGCGGGCATCTGGTTC-3' |
